# Pregnancy outcomes in patients receiving assisted reproductive therapy with systemic lupus erythematosus: a multi-center retrospective study

**DOI:** 10.1186/s13075-023-02995-y

**Published:** 2023-01-25

**Authors:** Minxi Lao, Peiyin Dai, Guangxi Luo, Xing Yang, Miaoguan Peng, Yuyi Chen, Yanfeng Zhan, Zhongping Zhan, Dongying Chen

**Affiliations:** 1 Department of Rheumatology, The First Affiliated Hospital of Sun Yat-Sen University, Guangzhou, China; 2 Department of Geriatrics, The First Affiliated Hospital of Sun Yat-Sen University, Guangzhou, China; 3 Center of Reproductive Medicine, The Sixth Affiliated Hospital of Sun Yat-Sen University, Guangzhou, China; 4 Department of Endocrinology, The Third Affiliated Hospital of Guangzhou Medical University, Guangzhou, China; 5 Department of Obstetrics, The Third Affiliated Hospital of Guangzhou Medical University, Guangzhou, China; 6 Department of Obstetrics, The First Affiliated Hospital of Sun Yat-Sen University, Guangzhou, China

**Keywords:** Assisted reproductive therapy, Systemic lupus erythematosus, Pregnancy, Anti-phospholipid antibody, Ooutcome

## Abstract

**Objectives:**

To evaluate the safety, efficacy, and maternal and fetal outcomes of assisted reproductive therapy (ART) in systemic lupus erythematosus (SLE).

**Methods:**

Patients from three tertiary hospitals from Guangzhou, China followed-up from 2013 to 2022 were included retrospectively. Patients with planned or unplanned natural pregnancy were chosen as controls. ART procedure and pregnancy outcomes were recorded and compared.

**Results:**

A total of 322 ART cycles in 142 women were analyzed. Sixty-six intrauterine pregnancies out of 72 clinical pregnancies yielded 65 live infants, including 5 pairs of twins. The clinical pregnancy rate was 46.5% (66/142). The mean age at the first clinical pregnancy was 34.0 ± 3.8 years. The median (interquartile range, IQR) disease course was 42.5 (25, 84.8) months. Twenty-seven (40.9%) of them had a history of adverse pregnancy. Primary infertility occurred in 20 (30.3%) patients. Obstruction of fallopian tubes (17/66, 25.8%) and premature ovarian failure (9/66, 13.6%) were the leading causes for infertility. Ovulation induction therapy (OIT) were conducted in 60 (83.3%) pregnancies, and no ovarian hyperstimulation syndrome (OHSS) or thrombosis was observed. The leading maternal adverse pregnancy outcomes (APOs) included premature delivery (21/66, 31.8%), gestational diabetes mellitus (GDM) (15/66, 22.7%), and disease flares (10/66, 15.2%). Spontaneous premature delivery (9/21, 42.9%) and preterm premature rupture of membranes (PPROM) (6/21, 28.6%) were the leading causes for premature delivery. Preeclampsia (19.0% vs 0%, *P* = 0.012) increased in premature delivery. Infants delivered prematurely were likely to be low-birth-weight (LBW)/very-low-birth-weight (VLBW) (81.0% vs 7.7%, *P* < 0.001). Disease flares were mild (4/10, 40.0%) or moderate (5/10, 50.0%), and developed during the second (3/10, 30.0%) or third (6/10, 60.0%) trimester with favorable outcomes. Fetal loss in ART (6/66, 9.1%) was primarily attributed to early spontaneous abortion (*n* = 5). The average delivery time was 36.8 ± 2.1 weeks of gestation. The average birth weight was 2653.5 ± 578.6 g. LBW infants accounted for 30.8% (20/65). No neonatal death or neonatal lupus occurred. The incidence of adverse pregnancy outcomes did not increase in patients with ART compared with planned pregnancy and reduced significantly compared with an unplanned pregnancy.

**Conclusion:**

The safety and efficacy of ART is assured in lupus patients with stable disease. Maternal and fetal APOs are comparable with planned pregnancy, with a relatively high incidence of premature delivery, GDM, and LBW infants.

## Introduction

Systemic lupus erythematosus (SLE) is a chronic autoimmune disease affecting multiple organs including the reproductive system. Most of the patients with SLE are at childbearing age. Benefit from the diagnostic and therapeutic advances, patients with SLE are no longer discouraged from pregnancy under certain circumstances. However, it has been estimated that more than 50% of the lupus patients have less children than desired [1]. Chronic inflammation in SLE causes dysfunction of the hypothalamic-pituitary-ovarian axis, auto-oophoritis, and abnormal uterine bleeding, resulting in menstrual irregularity and premature ovarian failure [2]. The presence of anti-phospholipid antibodies leads to microthrombosis and impairs the embryo implantation [3]. Toxicity secondary to immunosuppressants such as cyclophosphamide (CYC) represents a common driver of infertility across SLE. Besides, worries for disease flares contribute to the postponing of pregnancy.

Assisted reproductive therapy (ART) is a well-developed technique for infertility and is widely used in the general population. The application of ART in lupus patients remains to be a matter of ongoing debate. Worries lie in lupus flares and thrombotic events induced by ovulation induction therapy (OIT), and the uncertainty of pregnancy outcomes hinders the manipulation of ART. With the advances in our knowledge and treatment options, however, ART is carried out successfully in lupus patients. It is strongly recommended in women with uncomplicated rheumatic and musculoskeletal diseases whose disease is stable/quiescent [4].

Until recently, most studies of ART procedures pay little attention to maternal diagnosis. Studies of ART with interest in SLE report very small numbers. Herein, we conducted a multi-center retrospective study to evaluate the safety, efficacy, and complications of ART in SLE.

## Patients and methods

### Patients and data collection

A multi-center, observational, retrospective study was performed. Consecutive patients with SLE followed up during one or more cycles of ART in three tertiary hospitals from Guangzhou (i.e., the First Affiliated Hospital of Sun Yat-sen University, the Sixth Affiliated Hospital of Sun Yat-sen University, the Third Affiliated Hospital of Guangzhou Medical University) from January 2013 to October 2022 were included. Lupus patients with natural pregnancy during the same period were selected as controls and divided into planned pregnancy and unplanned pregnancy. SLE was diagnosed according to the 1997 ACR diagnostic criteria [5]. The data of clinical phenotype of lupus, obstetric history, outcome of pregnancies, obstetrical complications, perinatal complications of newborns, laboratory tests, and medical treatments before and during pregnancy were collected and reviewed by two rheumatologists (D.C and Z.Z).

### ART procedure

Woman meeting the following criteria were eligible for ART: (1) incapacity to fulfill pregnancy after a reasonable time (> 1 year) of sexual intercourse with no contraceptive measures taken, (2) no moderate or severe SLE flares for at least a year, (3) absence of major organ dysfunction, (4) oral prednisone ≤ 10 mg per day, (5) discontinuation of immunosuppressants including CYC, methotrexate, and mycophenolate mofetil for at least 6 months, for those who were taking leflunomide, wash-out therapy was administered and leflunomide was withdrawn for at least 6 months. All the patients were transferred to the reproductive centers after disease evaluation by rheumatologists. Patients were re-evaluated by the obstetricians for indications for ART. Those who received ART were followed by a multi-disciplinary team afterwards according to Chinese recommendations for perinatal care in high-risk women [6].

ART included ovarian stimulation, oocyte retrieval, in vitro fertilization (IVF), and transfer of the fertilized embryo into the uterus. Intrauterine insemination (IUI) was performed by injecting the sperm directly into the uterus. IVF/intracytoplasmic sperm injection (ICSI) was conducted by removing an oocyte from the ovaries, fertilizing it with semen outside the body, and then replacing it back to the womb. Preimplantation genetic testing (PGT) encompasses preimplantation genetic screening (PGS) and preimplantation genetic diagnosis (PGD). Briefly, embryos cultured in vitro were analyzed for a monogenic disease, and only disease-free embryos were transferred to the mother, to avoid the termination of pregnancy with an affected fetus. PGT was primarily applied to elderly pregnant women, women with recurrent miscarriage, women with recurrent transplant failure, parental chromosomal diseases, parental monogenic inherited diseases, and parental thalassemia. Clinical pregnancy, including intrauterine pregnancy and ectopic pregnancy, was confirmed with elevated serum human chorionic gonadotropin (hCG) level 14 days after transplantation and gestational sacs identified 4–6 weeks after transplantation by ultrasonography. Clinical pregnancy rate was defined as patients detected with fetal heartbeat divided by the total patients.

### Controlled OIT

Individualized OIT was performed by obstetricians, and the following protocols were used. Long protocol: Gonadotropin-releasing hormone (GnRH) agonist was administered in the midluteal phase. Obstetric ultrasound examination was performed, and the sex hormone levels (i.e., follicle-stimulating hormone (FSH), luteinizing hormone (LH), and estradiol) were detected 14 days after. Individualized injection with recombinant FSH was prescribed accordingly. Ultralong protocol: GnRH agonist was administered on day 2 of the menstrual period and repeated for 1–3 cycles. Ovarian stimulation was started by gonadotropin (Gn) 28 days after the last injection of GnRH. Antagonist protocol: Exogenous Gn was given on day 2 of the menstrual period, followed by antagonist four days later to prevent early ovulation. Other unconventional protocols: Modified natural cycle with a small amount of Gn. Mini-dose protocol with a small dose of Gn in a patient having low ovarian reserves.

### Definition

Primary infertility was defined as a couple that has never been able to conceive a pregnancy after a minimum of one year of attempting to do so through unprotected intercourse. Secondary infertility was defined as the inability to conceive or carry a pregnancy to term after having already successfully given birth to one or more children.

Planned pregnancy was defined according to Chinese recommendations for perinatal management in women with SLE [7]. Unplanned pregnancy included patients who became pregnant in active disease, withdrew medication without doctors’ guidance, and new onset of SLE during pregnancy.

Pregnancy trimesters were defined as follows: first trimester: up to 13^th^ weeks of gestation; second: from 14 to 26^th^ weeks; third: after 27^th^ week. SLE activity was measured by Systemic Lupus Erythematosus Pregnancy Disease Activity Index (SLEPDAI) during gestation. Mild disease activity was defined as SLEPDAI score 5 to 9, moderate disease activity as SLEPDAI score 10 to 14, and high disease activity as SLEPDAI score ≥ 15 [8]. The highest score was used in statistical analysis.

### Adverse pregnancy outcomes (APOs)

Maternal APOs: (1) Disease flare: worsening or new symptoms attributed to SLE. An SLE flare was established by an increase ≥ 3 in SLEPDAI [9]. (2) Pregnancy-induced hypertension (PIH): hypertension during pregnancy, which included gestational hypertension, preeclampsia, and eclampsia. Hypertension was defined as systolic blood pressure > 140 mmHg and/or diastolic blood pressure > 90 mmHg in sitting position in at least two consecutive measurements during pregnancy. (3) Preeclampsia: a new onset of hypertension with or without proteinuria after the 20^th^ week of gestation in a previously normotensive woman [10]. (4) Eclampsia: the occurrence of one or more generalized, tonic–clonic convulsions unrelated to other medical conditions in women with hypertensive disorder of pregnancy [9]. (5) Gestational diabetes mellitus (GDM): the onset or first recognition of diabetes occurred during pregnancy. Diabetes was confirmed by oral glucose tolerance test (OGTT) fasting plasma glucose ≥ 92 mg/dl (5.1 mmol/dl), or 1‑hour glucose in venous plasma ≥ 180 mg/dl (10.0 mmol/l), or OGTT 2‑hour glucose in venous plasma ≥ 153 mg/dl (8.5 mmol/l) [11]. (6) Fetal loss included spontaneous abortion, therapeutic abortion (artificial termination of pregnancy due to the exacerbation of lupus or obstetric complications), intrauterine fetal death (IUFD, death of a fetus due to the reasons other than chromosomal abnormalities, anatomic malformation, or congenital infection after 20 weeks of gestation), and neonatal death (death of a live infant within 28 days after birth). (7) Premature delivery: birth under 37 weeks gestational age, with further classification into late (34 weeks and above) and early (under 34 weeks) preterm births. (8) Preterm premature rupture of membranes (PPROM): the spontaneous rupture of membranes during pregnancy before the 37^th^ week of gestation.

Fetal APOs: (1) Intrauterine growth retardation (IUGR): birth weight below the 10^th^ percentile of the Chinese population according to gestational week at delivery and fetal gender. (2) Fetal distress: fetus hypoxia and acidosis which endangers the health of the fetus. (3) Low-birth-weight (LBW): infant with birth weight < 2500 g. (4) Very-low-birth-weight (VLBW): infant with birth weight < 1500 g.

### Statistical analysis

Statistical analysis was performed using SPSS 25.0 (SPSS Inc, Chicago, IL, USA). Normally distributed data were presented as mean ± standard deviation (SD), and non-normal variables were expressed as median (interquartile range, IQR). Categorical variables were presented as frequency and percentage. Comparison among groups was evaluated with chi-square test or Fisher exact test for categorical variables and one-way ANOVA for continuous variables with normal distribution. Between-group comparison was evaluated using Student’s *t* test for continuous variables with normal distribution, and Mann–Whitney *U* test for continuous variables with non-normal distribution. A *P* value < 0.05 was considered statistically significant in comparison among the three groups. A *P* value < 0.016 was considered statistically significant in comparison between any two groups.

## Results

### Demographic data

A total of 322 ART cycles was performed in 142 consecutive patients. Finally, 66 patients ended in 72 clinical pregnancies, including 66 intrauterine pregnancies and 6 ectopic pregnancies. Clinical pregnancy rate was 46.5% (66/142). The mean age at the first clinical pregnancy was 34.0 ± 3.8 years. The median (IQR) disease course was 42.5 (25, 84.8) months.

Approximately 40.9% (27/66) of them had histories of adverse pregnancy, including spontaneous abortion (17/66, 25.8%), ectopic pregnancy (11/66, 16.7%), therapeutic abortion (7/66, 10.6%), and preeclampsia (1/66, 1.5%) (Table 1).

**Table 1:** Basic characteristics of patients with ART. Pa, comparisons among three groups. Pb, comparison between ART and planned pregnancy. Pc, comparison between ART and unplanned pregnancy. Pd, comparison between planned and unplanned pregnancy

	**ART (*n* = 66)**	**Planned (*n* = 214)**	**Unplanned (*n* = 66)**	**Pa**	**Pb**	**Pc**	**Pd**
**Basic characteristics**							
Age, mean ± SD	34.0 ± 3.8	31.3 ± 4.3	28.4 ± 5.1	< 0.001	< 0.001	< 0.001	< 0.001
Pre-pregnant BMI, kg/m^2^, mean ± SD	21.4 ± 2.3	20.4 ± 2.7	21.2 ± 2.3	0.006	0.007	0.618	0.030
Disease course, months, median (IQR)	42.5 (25, 84.8)	72 (41, 108)	33.5 (10.5, 63)	< 0.001	< 0.001	< 0.001	< 0.001
Primipara, *n* (%)	43 (65.2)	152 (71.0)	35 (53.0)	0.025	0.444	0.215	0.008
Previous pregnancies, mean ± SD	2.6 ± 1.6	2.1 ± 1.2	2.0 ± 1.3	0.012	0.007	0.020	0.562
**Obstetrical history**							
Incidence rate, *n* (%)	27 (40.9)	68 (31.8)	10 (15.2)	0.004	0.183	0.002	0.011
Spontaneous abortion, *n* (%)	17 (25.8)	33 (15.4)	5 (7.6)	0.016	0.066	0.009	0.148
Ectopic pregnancy, *n* (%)	11 (16.7)	4 (1.9)	0 (0)	< 0.001	< 0.001	0.001	0.576
Therapeutic abortion, *n* (%)	7 (10.6)	19 (8.9)	3 (4.5)	0.424			
Preeclampsia/eclampsia, *n* (%)	1 (1.5)	4 (1.9)	0 (0)	0.825			
Stillbirth,* n* (%)	0 (0)	9 (4.2)	1 (1.5)	0.138			
Premature delivery, *n* (%)	0 (0)	8 (3.7)	2 (3.0)	0.277			
Neonatal death, *n* (%)	0 (0)	2 (0.9)	0 (0)	1.000			
**Laboratory test**							
ANA, *n* (%)	59 (89.4)	187 (87.4)	64 (97.0)	0.088			
Anti-dsDNA, *n* (%)	15 (22.7)	114 (53.3)	48 (72.7)	< 0.001	< 0.001	< 0.001	0.007
Anti-SSA, *n* (%)	16 (24.2)	93 (43.5)	41 (62.1)	< 0.001	0.006	< 0.001	0.011
Anti-SSB, *n* (%)	8 (12.1)	20 (9.3)	11 (16.7)	0.262			
aCL-IgG, *n* (%)	2 (3.0)	10 (4.7)	3 (4.5)	0.933			
aCL-IgM, *n* (%)	0 (0)	3 (1.4)	4 (6.1)	0.058			
β2-GPI-IgG, *n* (%)	2 (3.0)	3 (1.4)	2 (3.0)	0.356			
β2-GPI-IgM, *n* (%)	3 (4.5)	0 (0)	1 (1.5)	0.013			
LA, *n* (%)	1 (1.5)	4 (1.9)	3 (4.5)	0.473			
**Pre-pregnant treatment**							
Glucocorticoids, *n* (%)	26 (39.4)	200 (93.5)	26 (39.4)	< 0.001	< 0.001	1.000	< 0.001
Immunosuppressants, *n* (%)	4 (6.1)	38 (17.8)	8 (12.1)	0.055	0.028	0.365	0.344
CsA, *n* (%)	4 (6.1)	22 (10.3)	4 (6.1)	0.295			
TAC, *n* (%)	0 (0)	7 (3.3)	1 (1.5)	0.371			
AZA, *n* (%)	0 (0)	9 (4.2)	0 (0)	0.050			
HCQ, *n* (%)	57 (86.4)	176 (82.2)	20 (30.3)	< 0.001	0.461	0.461	< 0.001
LDA, *n* (%)	10 (15.2)	35 (16.4)	1 (1.5)	0.007	0.852	0.009	0.003
LMWH, *n* (%)	7 (10.6)	11 (5.1)	0 (0)	0.014	0.148	0.013	0.072

*Abbreviation*: *ART* Assisted reproductive therapy, *AZA* Azathioprine, *BMI* Body mass index, *CsA* Cyclosporin A, *HCQ* Hydroxychloroquine, *IQR* Interquartile range, *LA* Lupus anticoagulant, *LDA* Low-dose aspirin, *LMWH* Low molecular weight heparin, *SD* Standard deviation, *TAC* Tacrolimus

The positive of aCL-IgG was 3.0% (2/66). The positive of β2-GPI-IgG and β2-GPI-IgM were 3.0% (2/66) and 4.5% (3/66), respectively. The positive of lupus anticoagulants (LA) was 1.5% (1/66). Pre-pregnant treatment included glucocorticoids (26/66, 39.4%), cyclosporin A (CsA) (4/66, 6.1%), hydroxychloroquine (57/66, 86.4%), low-dose aspirin (LDA) (10/66, 15.2%), and low molecular weight heparin (LMWH) (7/66, 10.6%).

### Manipulation of ART

Primary infertility occurred in 20 (30.3%) patients, while secondary infertility in 46 (69.7%) patients. Underlying causes were shown in Table 2. Obstruction of fallopian tubes (17/66, 25.8%) and premature ovarian failure (9/66, 13.6%) were the leading. Two patients (3.0%) had anti-phospholipid syndrome (APS).

**Table 2:** Causes for ART

Causes for ART	*n* = 66
Obstruction of fallopian tubes, *n* (%)	17 (25.8)
Premature ovarian failure, *n* (%)	9 (13.6)
Endometriosis, *n* (%)	7 (10.6)
Uterine malformation, *n* (%)	2 (3.0)
APS, *n* (%)	2 (3.0)
Male factor, *n* (%)	7 (10.6)
Mixed,* n* (%)	12 (18.2)
Unexplained, *n* (%)	11 (16.7)

*Abbreviation*: *APS* Anti-phospholipid syndrome, *ART* Assisted reproductive therapy

OIT was conducted in 60 (83.3%) pregnancies, including long protocol (27/72, 37.5%), mini-dose protocol (12/72, 16.7%), modified natural cycle (12/72, 16.7%), antagonist protocol (7/72, 9.7%), and ultralong protocol (2/72, 2.8%). IVF was performed in 58 (80.6%) pregnancies, ICSI in 7 (9.7%), IUI in 2 (2.8%), and PGT in 5 (6.9%). Neither ovarian hyperstimulation syndrome (OHSS) nor thrombotic event was observed.

### Maternal outcomes

Sixty (83.3%) pregnancies ended in successful delivery. Cesarean section was conducted in 42 pregnancies (42/66, 63/6%). Maternal APOs were shown in Table 3. Premature delivery was a complication with the highest incidence (21/66, 31.8%). The causes for premature delivery included spontaneous premature delivery (9/21, 42.9%), PPROM (6/21, 28.6%), obstetric problems (4/21, 19.0%), and mixed (2/21, 9.5%). The average delivery time of premature delivery was 36.8 ± 2.2 weeks of gestation, including 4 patients (19.0%) with early premature delivery and 17 patients (81.0%) with late premature delivery.

**Table 3:** Maternal outcomes in ART. Pa, comparisons among three groups. Pb, comparison between ART and planned pregnancy. Pc, comparison between ART and unplanned pregnancy. Pd, comparison between planned and unplanned pregnancy

	**ART (*n* = 66)**	**Planned (*n* = 214)**	**Unplanned (*n* = 66)**	**Pa**	**Pb**	**Pc**	**Pd**
**Mode of delivery**							
Natural labor, *n* (%)	18 (27.3)	72 (33.6)	11 (16.7)	0.028	0.368	0.207	0.009
Cesarean section, *n* (%)	42 (63.6)	126 (58.9)	12 (18.2)	< 0.001	0.566	< 0.001	< 0.001
**Outcomes**							
GDM, n (%)	15 (22.7)	16 (7.5)	1 (1.5)	< 0.001	0.001	< 0.001	0.084
Disease flares, *n* (%)	10 (15.2)	23 (10.7)	52 (78.8)^a^	< 0.001	0.382	< 0.001	< 0.001
PPROM, *n* (%)	9 (13.6)	54 (25.2)	2 (3.0)	< 0.001	0.063	0.055	< 0.001
Preeclampsia, *n* (%)	5 (7.6)	13 (6.1)	0 (0)	0.056			
Gestational hypertension, *n* (%)	3 (4.5)	7 (3.3)	2 (3.0)	0.913			
Eclampsia, *n* (%)	1 (1.5)	14 (6.5)	4 (6.1)	0.313			
Premature delivery, *n* (%)	21 (31.8)	48 (22.4)	14 (21.2)	0.259			
Fetal loss, *n* (%)	6 (9.1)	16 (7.5)	43 (65.2)	< 0.001	0.794	< 0.001	< 0.001
Twins, pairs, *n* (%)	5 (7.6)	3 (1.4)	0 (0)	0.019	0.020	0.058	1.000

*Abbreviation*: *ART* Assisted reproductive therapy, *GDM* Gestational diabetes mellitus, *PIH* Pregnancy-induced hypertension, *PPROM* Preterm premature rupture of membranes, *SLE* Systemic lupus erythematosus

^a^Including 19 pregnancies with new onset of SLE after pregnancy

The incidence of GDM ranked second (15/66, 22.7%). Among 15 patients with GDM, the mean age was 37.0 ± 4.5 years. The mean pre-pregnant body mass index (BMI) was 22.1 ± 2.1 kg/m^2^. Fetal loss occurred in two pregnancies (13.3%). Fourteen infants were born, including a pair of twins and three LBW infants.

Ten patients (15.2%) experienced disease flares. Most of the severity was mild (4/10, 40.0%) or moderate (5/10, 50.0%). Active disease tended to develop during the second (3/10, 30.0%) or third (6/10, 60.0%) trimester. Lupus nephritis (8/10, 80.0%) was the main manifestation, followed by thrombocytopenia (5/10, 50.0%). Nine out of ten (90.0%) delivered live infants (Table 4).

**Table 4:** Manifestation of disease flares. Pa, comparisons among three groups. Pb, comparison between ART and planned pregnancy. Pc, comparison between ART and unplanned pregnancy. Pd, comparison between planned and unplanned pregnancy

	**ART (*n* = 10)**	**Planned (*n* = 23)**	**Unplanned (*n* = 52)**	**Pa**	**Pb**	**Pc**	**Pd**
**Disease activity**							
Mild, *n* (%)	4 (40.0)	23 (100)	13 (25.0)	< 0.001	0.004	0.440	< 0.001
Moderate, *n* (%)	5 (50.0)	0 (0)	11 (21.2)	0.001	0.001	0.107	0.015
Severe, *n* (%)	1 (10.0)	0 (0)	28 (53.8)	< 0.001	0.303	0.015	< 0.001
**Time of disease flares**							
First trimester, *n* (%)	1 (10.0)	6 (26.1)	28 (53.8)	0.008	0.397	0.015	0.043
Second trimester, *n* (%)	3 (30.0)	11 (47.8)	20 (38.5)	0.580			
Third trimester, *n* (%)	6 (60.0)	6 (26.1)	1 (1.9)	< 0.001	0.114	< 0.001	0.003
Puerperium, *n* (%)	0 (0)	0 (0)	3 (5.8)	0.691			
**Manifestations**							
Nephritis, *n* (%)	8 (80.0)	18 (78.3)	28 (53.8)	0.050			
Proteinuria, *n* (%)	8 (80.0)	17 (73.9)	28 (53.8)	0.125			
Hematuria, *n* (%)	2 (20.0)	1 (4.3)	3 (5.8)	0.716			
Pyuria, *n* (%)	1 (10.0)	0 (0)	0 (0)	0.182			
Thrombocytopenia, *n* (%)	5 (50.0)	4 (17.4)	19 (36.5)	0.329			
Leukopenia, *n* (%)	1 (10.0)	2 (8.7)	4 (7.7)	0.182			
Rash, *n* (%)	1 (10.0)	0 (0)	11 (21.2)	0.046			
Hemolytic anemia, *n* (%)	0 (0)	2 (8.7)	2 (3.8)	0.716			
NP-SLE, *n* (%)	0 (0)	1 (4.3)	3 (5.8)	1.000			
Arthritis, *n* (%)	0 (0)	2 (8.7)	6 (11.5)	0.675			

*Abbreviation*: *ART* Assisted reproductive therapy, *NP-SLE* Neuropsychiatric-systemic lupus erythematosus

### Fetal outcomes

Sixty-five live infants, including 5 pairs of twins, were born from 60 intrauterine pregnancies. The live birth rate was 83.3% (60/72). The average delivery time was 36.8 ± 2.1 weeks of gestation. The average birth weight was 2653.5 ± 578.6 g. The incidence of fetal APOs were shown in Table 5. Ten infants (15.4%) were transferred to the intensive care unit (ICU) after delivery. No neonatal lupus or fetal atrioventricular block was observed.

**Table 5:** Fetal outcomes in ART. Pa, comparisons among three groups. Pb, comparison between ART and planned pregnancy. Pc, comparison between ART and unplanned pregnancy. Pd, comparison between planned and unplanned pregnancy

	**ART (*n* = 65)**	**Planned (*n* = 201)**	**Unplanned (*n* = 23)**	**Pa**	**Pb**	**Pc**	**Pd**
**Outcomes**							
Time of delivery, weeks, mean ± SD	36.8 ± 2.1	37.1 ± 2.0	34.8 ± 3.1	< 0.001	0.267	0.001	< 0.001
Birth weight, mean ± SD	2653.5 ± 578.6	2703.3 ± 491.3	2298.3 ± 762.5	0.003	0.498	0.023	0.001
IUGR, *n* (%)	5 (7.7)	17 (8.5)	4 (17.4)	0.383			
Fetal distress, *n* (%)	5 (7.7)	32 (15.9)	3 (13.0)	0.233			
LBW infants, *n* (%)	20 (30.8)	51 (25.4)	7 (30.4)	0.641			
VLBW infants, *n* (%)	2 (3.1)	5 (2.5)	5 (21.7)	0.001	0.680	0.012	0.001
Neonatal hypoglycemia, *n* (%)	2 (3.1)	2 (1.0)	1 (4.3)	0.188			
Neonatal pneumonia, *n* (%)	3 (4.6)	3 (1.5)	1 (4.3)	0.702			
NRDS, *n* (%)	5 (7.7)	7 (3.5)	1 (4.3)	0.621			
Neonatal pathological jaundice, *n* (%)	11 (16.9)	29 (14.4)	2 (8.7)	0.702			
Malformation, *n* (%)	6 (9.2)	12 (6.0)	1 (4.3)	0.621			
PDA, *n* (%)	2 (3.1)	9 (4.5)	2 (8.7)	0.501			
PFO, *n* (%)	7 (10.8)	24 (11.9)	1 (4.3)	0.707			
ASD, *n* (%)	4 (6.2)	5 (2.5)	1 (4.3)	0.280			
Transfer to ICU, *n* (%)	10 (15.4)	70 (34.8)	8 (34.8)	0.011	0.003	0.070	1.000
**Laboratory tests**							
ANA, *n* (%)	12 (18.5)	24 (11.9)	4 (17.4)	0.313			
Anti-dsDNA, *n* (%)	5 (7.7)	11 (5.5)	3 (13.0)	0.244			
Anti-SSA, *n* (%)	6 (9.2)	12 (6.0)	3 (13.0)	0.265			
Anti-SSB, *n* (%)	2 (3.1)	0 (0)	1 (4.3)	0.028	0.059	1.000	0.103

*Abbreviation*: *ART* Assisted reproductive therapy, *ASD* Atrial septal defect, *ICU* Intensive care unit, *LBW* Low-birth-weight, *IUGR* Intrauterine growth retardation, *NRDS* Neonatal respiratory distress syndrome, *PDA* Patent ductus arteriosus, *PFO* Patent foramen ovale, *SD* Standard deviation, *VLBW* Very-low-birth-weight

Infants of LBW accounted for 30.8% (20/65), and infants of VLBW accounted for 3.1% (2/65). Approximately 63.6% (14/22) of the LBW/VLBW infants were born prematurely. Six VLBW and 2 LBW infants were from twin gestation (8/22, 36.4%). The incidence of fetal APOs such as neonatal pathological jaundice (45.5% vs 2.3%, *P* < 0.001), neonatal pneumonia (13.6% vs 0%, *P* = 0.035), and neonatal respiratory distress syndrome (NRDS) (13.6% vs 0%, *P* = 0.035) increased significantly in LBW/VLBW infants. Nearly half of the LBW/VLBW infants (10/22, 45.5%) were transferred to ICU, compared to 7.0% (3/43) in term infants (*P* = 0.001).

### Comparison of pregnancy outcomes between patients with or without premature delivery

Considering premature delivery was the most common APO, we further compared premature delivery with term delivery. As shown in Table 6, preeclampsia (19.0% vs 0%, *P* = 0.012) occurred more frequently in premature delivery. Besides, the incidence of LBW infants increased significantly in premature delivery (81.0% vs 7.7%, *P* < 0.001).

**Table 6:** Comparison between premature and term delivery

	**Premature delivery (** ***n*** ** = 21)**	**Term delivery (** ***n*** ** = 39)**	***P*** ** value**
**Maternal APOs**			
Disease flares, *n* (%)	3 (14.3)	4 (10.3)	0.687
GDM, *n* (%)	4 (19.0)	9 (23.1)	1.000
Preeclampsia, *n* (%)	4 (19.0)	0 (0)	0.012
Gestational hypertension, *n* (%)	1 (4.8)	2 (5.1)	1.000
Eclampsia, *n* (%)	1 (4.8)	0 (0)	0.350
**Fetal APOs**			
LBW, *n* (%)	17 (81.0)	3 (7.7)	< 0.001
VLBW, *n* (%)	0 (0)	2 (5.1)	0.537
Fetal distress, *n* (%)	2 (9.5)	3 (7.7)	1.000
IUGR, *n* (%)	2 (9.5)	3 (7.7)	1.000

*Abbreviation*: *APO* Adverse pregnancy outcome, *ART* Assisted reproductive therapy, *GDM* Gestational diabetes mellitus, *IUGR* Intrauterine growth retardation, *LBW* Low-birth-weight, *PIH* Pregnancy-induced hypertension, *VLBW* Very-low-birth-weight

### Comparison of pregnancy outcomes in ART, planned pregnancy, and unplanned pregnancy

In the comparison with planned pregnancies (*n* = 214) and unplanned pregnancies (*n* = 66), patients receiving ART were older (34.0 vs 31.1 vs 28.4 years, *P* < 0.001), and more likely to complicated with history of adverse pregnancy (40.9% vs 31.8% vs 15.2%, *P* = 0.004). Patients receiving ART were likely to develop GDM (22.7% vs 7.5% vs 1.5%, *P* < 0.001). Disease flares (15.2% vs 10.7% vs 78.8%, *P* < 0.001) and fetal loss (9.1% vs 7.5% vs 65.2%, *P* < 0.001) decreased significantly in ART. As for disease flares, patients receiving ART or with planned pregnancy usually had mild-to-moderate activity, while patients with unplanned pregnancy usually had severe activity (10.0% vs 0% vs 53.8%, *P* < 0.001). Disease flares were likely to occur during the second or third trimester in ART and planned pregnancy, and it occurred during the first or second trimester in unplanned pregnancy. The manifestation of active disease was similar in the three groups. However, organ involvement except for lupus nephritis and thrombocytopenia seemed to increase in unplanned pregnancy.

Reasons for fetal loss in ART included early spontaneous abortion (*n* = 5), and disease flare complicated with severe preeclampsia (*n* = 1). Reasons for fetal loss in planned pregnancy including stillbirth (*n* = 6), disease flares (*n* = 5), serious deformity (*n* = 2), early spontaneous abortion (*n* = 2), and uterine rupture (*n* = 1). Reasons for fetal loss in unplanned pregnancy included disease flare (*n* = 39), therapeutic abortion due to drug toxicity (*n* = 2), stillbirth (*n* = 1), and early spontaneous abortion (*n* = 1).

Time of delivery was earlier in unplanned pregnancy (36.8 vs 37.1 vs 34.8 weeks, *P* < 0.001), although premature delivery did not differ. The average birth weight was the lightest in unplanned pregnancy (2653.5 g vs 2703.3 g vs 2298.3 g, *P* = 0.003). Other fetal complications and the presence of auto-antibodies did not differ among groups.

## Discussion

We report the safety and pregnancy outcomes of ART in lupus patients with stable disease. Nearly half of the women (46.5%) receiving ART ended in clinical pregnancy, and 83.3% of the pregnancies ended in successful delivery. No OHSS or thrombotic event was reported. Only a few patients experienced disease flares. No neonatal lupus, fetal atrioventricular block or neonatal death occurred. Pregnant outcomes in lupus patients undergoing ART are favorable, although premature delivery, GDM, and LBW infants remain to be important issues.

The efficacy of ART in terms of clinical pregnancy rate in our study (46.5%) is comparable with that in the general population from the same center (57.1%) [12]. In previous studies, nearly 41.4% of patients with connective tissue diseases (CTD) became clinically pregnant via ART [13]. Previous and current findings suggest that ART is an effective method for infertility in patients with SLE. Timing to start ART is crucial. The optimal time-point for ART in SLE is not determined. In the current study, the disease was stable in all patients for more than 1 year. In other centers, patients were considered suitable for ART until the disease is stable for at least half a year [14]. Balancing the risk of disease flares and obstetrical complications, we consider patients with disease remission for at least one year as potential candidates for ART. However, the comparison of pregnancy outcomes in patients with different stable courses remains to be elucidated.

OHSS induced by OIT is one of the major concerns that hinder the implementation of ART in SLE. The incidence, however, does not increase in CTD (~ 3% of cycles) compared with the general population (3–8% of cycles) [15, 16]. Individualized stimulation therapy is essential in taking the causes for infertility and the level of sex hormones into consideration. It’s suggested OIT should only be used in true infertility [17]. GnRH antagonist program is relatively safe since it avoids the use of hCG which may induce high estradiol concentrations [16, 18]. In our study, no case of OHSS was observed. It further assured OIT does not confer additional risk in SLE.

A few of the patients experienced maternal or fetal APOs, despite that disease activity was well controlled before ART. Premature delivery, GDM, and disease flares were common. Among 72 pregnancies, 31.8% of them had premature infants. It exceeds that in the general Chinese population (6.2–7.2%) [19]. The incidence of premature delivery varies from 20 to 50% in other studies [17, 18, 21]. The reasons, however, were not well-presented. Nearly half of the pregnancies (42.9%) in our study were spontaneous preterm, and one fifth (28.6%) were attributed to PPROM. Of note, 19.0% of the women with premature delivery experienced preeclampsia. The incidence of LBW infants was also increased in premature delivery. Our results suggest that efforts to ensure full-term birth is of clinical significance.

The incidence of GDM in the ART group increased significantly (22.7%), compared with planned pregnancy (7.5%) and unplanned pregnancy (1.5%). In a European study recruiting 60 women with rheumatic disease undergoing ART, GDM occurred in 8.7% of the pregnancies. GDM occurs in 3.0–21.2% of the Asian pregnant women [20]. Traditional risk factors include high pre-pregnant BMI, advanced maternal age, personal or family history of diabetes, twin pregnancy, and genetic susceptibility [11]. ART emerges as another predisposing factor for GDM. In ART pregnancy, the increasing level of estrogen is associated with the incidence of GDM [21]. The average age of patients with GDM in our series was relatively old (37.0 ± 4.5), while pre-pregnant BMI was normal. The incidence of fetal loss in women complicating with GDM was high (13.3%). These results suggest that blood glucose needs to be supervised closely and early recognition of GDM might reduce fetal loss.

Disease flares (15.2%) in ART are as frequent as previously published (13–14.8%) [17, 18]. The incidence is in line with planned pregnancy (10.7%), and lower than that in unplanned pregnancy (78.8%). Maternal and fetal outcomes were comparable between patients with or without disease flares. One of the reasons could be that disease flares were usually mild-to-moderate and occurred during the second or third trimester. Nephritis (80.0%) was the major manifestation, followed by thrombocytopenia (50.0%). Generally, the pattern of disease flares did not differ between ART and planned pregnancy, while that in unplanned pregnancy involved various systems. Although a few patients experience the risk of disease flare during ART, the outcomes are usually satisfactory.

The occurrence of fetal loss did not increase in ART (9.1% in ART vs 7.5% in planned pregnancy vs 65.2% in unplanned pregnancy), and early spontaneous abortion is the leading cause. Only one fetal loss was attributed to disease flares. Early spontaneous abortion appears to be the most common cause and the rate ranges from 22 to 63% in the general patients after ART [22]. Many factors are attributable, such as advancing maternal age and genetic defects. Our results suggest a fetal loss is probably determined by obstetrical factors rather than disease flares in ART in SLE.

LBW/VLBW infants are common fetal complications, accounting for 33.9% in total. More than two-thirds (63.6%) of them were born prematurely. ART-conceived pregnancies have a higher incidence of LBW compared with all births (25.5% vs 8.1%), mainly due to multiple gestation [23]. We notice the proportion of LBW was slightly higher in ART than planned pregnancy, although no statistical significance was reached. VLBW infants counted for a very small number (3.1%). However, VLBW was frequent in unplanned pregnancy (21.7%). A higher proportion of LBW/VLBW infants were transferred to ICU. Efforts must be devoted to reducing the incidence of LBW/VLBW in order to minimize subsequent adverse outcomes.

Our study had a certain weakness. Few patients in our study were positive for aPL or LA. These patients were probably not considered for ART initially. The low positive of aPL hinders further analysis on the relation between aPL and APOs. However, previous studies showed that ART is safe even in patients with APS, and aPL does not increase the risk of thrombotic events and has no impact on pregnancy outcomes in ART [18]. In addition, the efficacy in different ART procedures or OIT was not analyzed due to the limited number of patients included.

ART is safe and yields satisfactory outcomes in lupus patients with stable disease for more than one year under close surveillance by a multidisciplinary team. Maternal and fetal APOs are comparable with planned pregnancy and reduced significantly compared with unplanned pregnancy. The incidences of premature delivery, GDM, and LBW infants, however, are still high.

## Data Availability

The raw data supporting the conclusions of this article will be made available by the authors, without undue reservation.

## Abbreviations

APO: Adverse pregnancy outcome
APS: Anti-phospholipid syndrome
ART: Assisted reproductive therapy
ASD: Atrial septal defect
AZA: Azathioprine
BMI: Body mass index
CsA: Cyclosporin A
CTD: Connective tissue diseases
CYC: Cyclophosphamide
FSH: Follicle-stimulating hormone
GDM: Gestational diabetes mellitus
Gn: Gonadotropin
GnRH: Gonadotropin-releasing hormone
hCG: Human chorionic gonadotropin
HCQ: Hydroxychloroquine
ICSI: Intracytoplasmic sperm injection
ICU: Intensive care unit
IQR: Interquartile range
IUFD: Intrauterine fetal death
IUGR: Intrauterine growth retardation
IUI: Intrauterine insemination
IVF: In vitro fertilization
LA: Lupus anticoagulants
LBW: Low-birth-weight
LDA: Low-dose aspirin
LH: Luteinizing hormone
LMWH: Low molecular weight heparin
NP-SLE: Neuropsychiatric-systemic lupus erythematosus
NRDS: Neonatal respiratory distress syndrome
OHSS: Ovarian hyperstimulation syndrome
OGTT: Oral glucose tolerance test
OIT: Ovulation induction therapy
PDA: Patent ductus arteriosus
PFO: Patent foramen ovale
PIH: Pregnancy-induced hypertension
PGD: Preimplantation genetic diagnosis
PGS: Preimplantation genetic screening
PGT: Preimplantation genetic testing
PPROM: Preterm premature rupture of membranes
SD: Standard deviation
SLE: Systemic lupus erythematosus
SLEPDAI: Systemic Lupus Erythematosus Pregnancy Disease Activity Index
TAC: Tacrolimus
VLBW: Very-low-birth-weight

## References

[1] Clowse M, Chakravarty E, Costenbader K, Chambers C, Michaud K (2012). Effects of infertility, pregnancy loss, and patient concerns on family size of women with rheumatoid arthritis and systemic lupus erythematosus. Arthritis Care Res (Hoboken).

[2] Oktem O, Yagmur H, Bengisu H, Urman B (2016). Reproductive aspects of systemic lupus erythematosus. J Reprod Immunol.

[3] Hunt BJ, Khamashta MA (2000). Antiphospholipid antibodies and the endothelium. Curr Rheumatol Rep.

[4] Sammaritano LR, Bermas BL, Chakravarty EE, Chambers C, Clowse MEB, Lockshin MD (2020). 2020 American College of Rheumatology Guideline for the Management of Reproductive Health in Rheumatic and Musculoskeletal Diseases. Arthritis Rheumatol.

[5] Hochberg MC (1997). Updating the American College of Rheumatology revised criteria for the classification of systemic lupus erythematosus. Arthritis Rheum.

[6] Obstetrics and Gynecology Group of Chinese Medical Association. Chinese recommendations for perinatal care in high-risk women (1st edition). Chin J Obstet Gynecol. 2011;46(2):150–3. Chinese. 10.3760/cma.j.issn.0529-567x.2011.02.018.

[7] The SLE study group of China. Chinese recommendations for perinatal management in women with SLE. Natl Med J China. 2015;95(14):1056–60. Chinese. 10.3760/cma.j.issn.0376-2491.2015.14.005.

[8] Buyon J, Kalunian K, Ramsey-Goldman R, Petri M, Lockshin M, Ruiz-Irastorza G (1999). Assessing disease activity in SLE patients during pregnancy. Lupus.

[9] Ruperto N, Hanrahan LM, Alarcón GS, Belmont HM, Brey RL, Brunetta P (2011). International consensus for a definition of disease flare in lupus. Lupus.

[10] Brown M, Lindheimer M, Swiet M, Assche A, Moutquin J. The classification and diagnosis of the hypertensive disorders of pregnancy: statement from the International Society for the Study of Hypertension in Pregnancy (ISSHP). Hypertens Pregnancy. 2001; 20(1): IX-XIV. 10.1081/PRG-100104165. 10.1081/PRG-100104165 12044323

[11] Chiefari E, Arcidiacono B, Foti D, Brunetti A (2017). Gestational diabetes mellitus: an updated overview. J Endocrinol Invest.

[12] Fan J, Zhong Y, Chen C (2017). Impacts of Anti-dsDNA Antibody on In Vitro Fertilization-Embryo Transfer and Frozen-Thawed Embryo Transfer. J Immunol Res.

[13] Reggia R, Andreoli L, Sebbar H, Canti V, Ceccarelli F, Favaro M, et al. An observational multicentre study on the efficacy and safety of assisted reproductive technologies in women with rheumatic diseases. Rheumatol Adv Pract. 2019;3(1):rkz005. 10.1093/rap/rkz005. 10.1093/rap/rkz005 PMC6649948 31431993

[14] Wechsler B, Le Thi Huong D, Vauthier-Brouzes D, Lefebvre G, Gompel A, Piette JC. Can we advise ovulation induction in patients with SLE? Scand J Rheumatol Suppl. 1998;107:53–9. 10.1080/03009742.1998.11720762. 10.1080/03009742.1998.11720762 9759134

[15] Nastri CO, Teixeira DM, Moroni RM, Leitao VM, Martins WP (2015). Ovarian hyperstimulation syndrome: pathophysiology, staging, prediction and prevention. Ultrasound Obstet Gynecol.

[16] Guballa N, Sammaritano L, Schwartzman S, Buyon J, Lockshin M (2000). Ovulation induction and in vitro fertilization in systemic lupus erythematosus and antiphospholipid syndrome. Arthritis Rheum.

[17] Huong D, Wechsler B, Vauthier-Brouzes D, Duhaut P, Costedoat N, Lefebvre G (2002). Importance of planning ovulation induction therapy in systemic lupus erythematosus and antiphospholipid syndrome: a single center retrospective study of 21 cases and 114 cycles. Semin Arthritis Rheum.

[18] Delvigne A, Rozenberg S (2002). Epidemiology and prevention of ovarian hyperstimulation syndrome (OHSS): a review. Hum Reprod Update.

[19] Blencowe H, Cousens S, Oestergaard M, Chou D, Moller A, Narwal R (2012). National, regional, and worldwide estimates of preterm birth rates in the year 2010 with time trends since 1990 for selected countries: a systematic analysis and implications. Lancet.

[20] Yuen L, Wong VW. Gestational diabetes mellitus: challenges for diferent ethnic groups. World J Diabetes. World J Diabetes. 2015;25;6(8):1024–32:10.4239/wjd.v6.i8.1024 10.4239/wjd.v6.i8.1024 PMC4515442 26240699

[21] Shiqiao H, Bei X, Yini Z, Lei J (2020). Risk factors of gestational diabetes mellitus during assisted reproductive technology procedures. Gynecol Endocrinol.

[22] Farr SL, Schieve LA, Jamieson DJ (2007). Pregnancy loss among pregnancies conceived through assisted reproductive technology, United States, 1999–2002. Am J Epidemiol.

[23] Reig A, Seli E (2019). The association between assisted reproductive technologies and low birth weight. Curr Opin Obstet Gynecol.

