# Correction: Pregnancy outcomes in patients receiving assisted reproductive therapy with systemic lupus erythematosus: a multi-center retrospective study

**DOI:** 10.1186/s13075-023-03004-y

**Published:** 2023-02-07

**Authors:** Minxi Lao, Peiyin Dai, Guangxi Luo, Xing Yang, Miaoguan Peng, Yuyi Chen, Yanfeng Zhan, Zhongping Zhan, Dongying Chen

**Affiliations:** 1 Department of Rheumatology, The First Affiliated Hospital of Sun Yat-Sen University, Guangzhou, China; 2 Department of Geriatrics, The First Affiliated Hospital of Sun Yat-Sen University, Guangzhou, China; 3 Center of Reproductive Medicine, The Sixth Affiliated Hospital of Sun Yat-Sen University, Guangzhou, China; 4 Department of Endocrinology, The Third Affiliated Hospital of Guangzhou Medical University, Guangzhou, China; 5 Department of Obstetrics, The Third Affiliated Hospital of Guangzhou Medical University, Guangzhou, China; 6 Department of Obstetrics, The First Affiliated Hospital of Sun Yat-Sen University, Guangzhou, China

**Correction: Arthritis Res Ther 25, 13 (2023)**

**https://doi.org/10.1186/s13075-023-02995-y**

Following publication of the original article [1], the authors reported that the e-mail address of corresponding author Zhongping Zhan is not correct. The correct e-mail address should be zhanzhp@mail.sysu.edu.cn.
